# Deep Learning-Assisted Autofocus for Aerial Cameras in Maritime Photography

**DOI:** 10.3390/jimaging12010031

**Published:** 2026-01-07

**Authors:** Haiying Liu, Yingchao Li, Shilong Xu, Haoyu Wang, Qiang Fu, Huilin Jiang

**Affiliations:** 1 School of Optoelectronic Engineering, Changchun University of Science and Technology, Changchun 130022, China; 2 Jilin Dongguang Group Co., Ltd., Changchun 130103, China

**Keywords:** UAV-mounted, visible-light camera, sea surface photography, autofocus, deep learning, defocus prediction

## Abstract

To address the unreliable autofocus problem of drone-mounted visible-light aerial cameras in low-contrast maritime environments, this paper proposes an autofocus system that combines deep-learning-based coarse focusing with traditional search-based fine adjustment. The system uses a built-in high-contrast resolution test chart as the signal source. Images captured by the imaging sensor are fed into a lightweight convolutional neural network to regress the defocus distance, enabling fast focus positioning. This avoids the weak signal and inaccurate focusing often encountered when adjusting focus directly on low-contrast sea surfaces. In the fine-focusing stage, a hybrid strategy integrating hill-climbing search and inverse correction is adopted. By evaluating the image sharpness function, the system accurately locks onto the optimal focal plane, forming intelligent closed-loop control. Experiments show that this method, which combines imaging of the built-in calibration target with deep-learning-based coarse focusing, significantly improves focusing efficiency. Compared with traditional full-range search strategies, the focusing speed is increased by approximately 60%. While ensuring high accuracy and strong adaptability, the proposed approach effectively enhances the overall imaging performance of aerial cameras in low-contrast maritime conditions.

## 1. Introduction

Visible-light cameras deployed on unmanned aerial vehicles (UAVs) have become a primary tool for maritime applications, including environmental monitoring, the reconnaissance of naval targets, and law enforcement. These systems are highly valued for their wide coverage and high spatial resolution [1]. However, optical imaging systems on UAVs encounter significant challenges when operating over vast oceanic areas. The low-pressure and low-temperature conditions encountered at high altitudes can induce physical changes in optical components, such as thermal expansion/contraction and variations in the refractive index, leading to defocus and severe degradation of image quality [2]. Compounding this problem, maritime scenes are typically characterized by sparse features, minimal texture, and inherently low contrast, posing considerable difficulties for image-based autofocus technologies.

Conventional autofocus methods for aerial cameras rely primarily on three types of techniques to counteract environmentally induced defocus. The first is the calibration and look-up table method, which uses environmental sensors (e.g., for temperature and pressure). This approach pre-calibrates a mapping relationship between these parameters and the optimal focal plane position. However, it suffers from poor generalizability and struggles to adapt to complex or uncalibrated external conditions [3]. The second method is electro-optical autocollimation. While potentially accurate, this technique requires complex optical paths and system configurations. Furthermore, its performance and the reliability of its feedback signal decrease significantly in low-contrast scenarios [4]. The third, and most widely researched, category is the search-based method, which relies on a full-frame image sharpness evaluation function. This method drives a motor to adjust the lens while computing a sharpness score in real time, seeking the position that maximizes this value. Unfortunately, in feature-sparse maritime environments, the sharpness evaluation function curve tends to be very flat and populated with numerous local extrema. This results in a computationally intensive, slow convergence process that is highly prone to settling on a local optimum, leading to autofocus failure [5].

The recent success of deep learning in computer vision offers a new paradigm for tackling complex regression and classification problems. Its powerful capabilities in image feature extraction and non-linear mapping show great potential for estimating optical system parameters, including the degree of defocus [6,7]. Some studies have begun exploring the use of convolutional neural networks (CNNs) to directly estimate blur kernels or defocus levels, providing novel ideas for rapid focus. Nevertheless, applying end-to-end deep learning models directly to the demanding task of airborne autofocus still presents challenges related to model complexity, computational resource constraints, and generalization across diverse scenarios. Recent advances in deep learning for optical system control have further demonstrated its efficacy in improving focusing speed and robustness in challenging environments [8]. Specific studies applying lightweight CNN architectures to defocus estimation have shown promising results in maintaining accuracy under computational constraints relevant to embedded systems [9]. Nevertheless, applying end-to-end deep learning models directly to the demanding task of airborne autofocus still presents challenges related to model generalizability across diverse, dynamic scenarios and strict platform resource limits.

To address the performance decline of traditional autofocus methods in low-contrast, low-texture maritime backgrounds—where weak image signals, flat evaluation functions, and local extrema often cause failures—this paper proposes a new autofocus system based on a built-in high-contrast signal source and deep learning assistance. By introducing a hybrid control architecture of “deep-learning coarse focusing + traditional search fine focusing,” the system transforms the challenging problem of focusing on sea scenes into a faster, more stable alignment task, leveraging a built-in high-resolution signal-to-noise test chart.

Specifically, the system takes images of the built-in test chart captured by the camera sensor as input. A lightweight convolutional neural network is used to regress and predict defocus distance, enabling fast, robust coarse focusing. This significantly reduces the range and time required for the subsequent fine search. Then, a hybrid strategy combining hill-climbing search and inverse correction is applied within the narrowed focus range. Using a sharpness evaluation function, the system accurately locates the optimal focal plane, forming a closed-loop control system.

Experiments demonstrate that this method substantially improves the speed and reliability of autofocus. It offers an effective technical approach for airborne cameras to achieve stable, high-quality imaging under complex maritime conditions.

## 2. System Architecture

### 2.1. Focusing Principle

The aerial camera objective lens adopts a dual-Gaussian quasi-symmetric structure. According to the Gaussian imaging theorem (Equation (1)), the relationships among the object distance u, image distance v, and focal length f are defined.

(1) $$\frac{1}{f}=\frac{1}{u}+\frac{1}{v}$$

**Figure 1:**
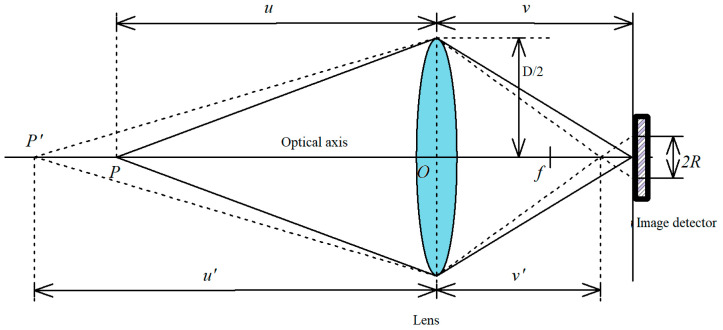
Simplified model of the optical imaging system.

A simplified model of the optical imaging system is shown in Figure 1, where u represents the ideal object distance, v represents the ideal image distance, u′ represents the actual object distance, v′ represents the actual image distance, f represents the lens focal length, D represents the diameter of the lens aperture, and R represents the radius of the circle of confusion.

During camera operation, an object located at the ideal object distance u results in light rays from a point P converging precisely onto the image plane, forming a sharp image on the detector. This state, where the detector’s focal plane coincides with the image plane at a distance v from the optical center, is defined as the in-focus position.

Conversely, if the object is situated at an alternate distance u′, the corresponding image point P′ is projected as a diffuse spot on the nominal image plane. The diameter of this spot, or the circle of confusion, is directly correlated with the degree of defocus: a larger spot indicates more severe defocus and a blurrier image. Since the extent of blur varies with deviation from the ideal image distance, the focal plane position must be adjusted to restore image sharpness. This adjustment process, in which the optical system is transitioned from a defocused state to a focused state, is termed focusing.

The focus evaluation function quantifies this phenomenon, typically yielding higher values for sharper images and peaking at the optimal focus. The autofocus algorithm presented in this paper integrates a novel deep learning approach with a traditional gradient-based search. The output of the algorithm controls the direction and step size of the focus drive motor to locate the peak of the sharpness function. A clear image can generally be attained when the system is adjusted to within half the depth of field.

The system uses a USAF 1951 standard-resolution test chart as the built-in target, with a maximum spatial frequency of 228 lp/mm and a pattern contrast greater than 80%. The target is illuminated by uniform LED backlight, with a color temperature of 5500 K and illumination uniformity exceeding 95%. This ensures a stable, high signal-to-noise ratio signal source for sharpness evaluation algorithms. The high-contrast edge structures of the target provide an ideal response characteristic for gradient-based sharpness evaluation functions.

### 2.2. System Design

**Figure 2:**
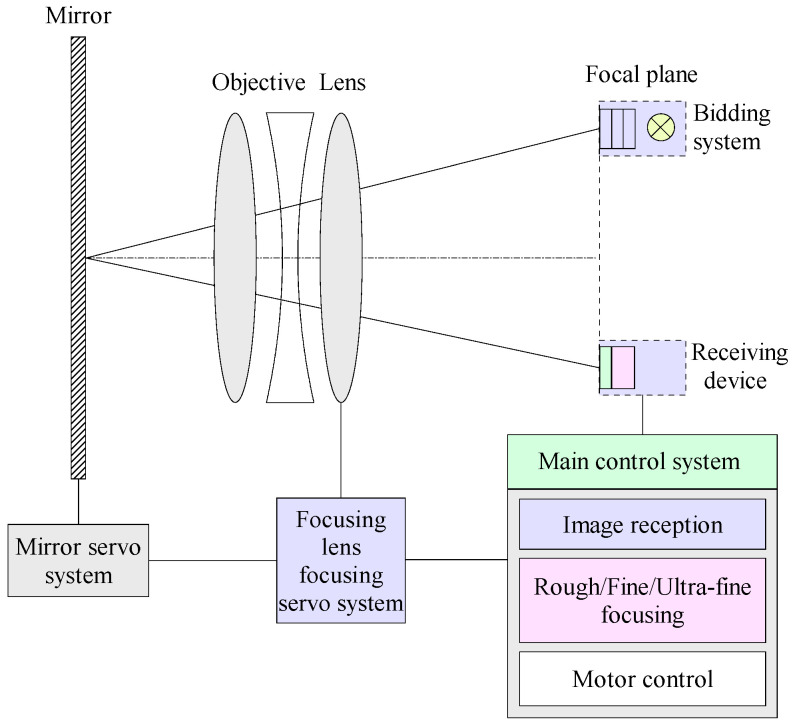
Block diagram of the built-in autofocus system.

This paper presents an autofocus system designed for aerial cameras in maritime photography. As illustrated in Figure 2, the system consists primarily of a projection unit (comprising an illumination source and a resolution target), an imaging sensor, a main control system, a focus servo mechanism, an internal focusing lens, and a mirror. A lightweight deep learning module is embedded within the main control system to predict the focal plane position during the coarse focusing stage.

The operational workflow is as follows: The projection unit generates an image of the built-in resolution target, which is projected through the objective lens and mirror onto the imaging sensor. The main control system first activates the deep learning module to analyze this image and infer an approximate optimal focal plane position. This prediction drives the servo mechanism to quickly position the lens near this location. The system subsequently switches to a conventional ramp search algorithm to perform fine and ultra-fine focusing, ultimately locking onto the precise optimal focal plane.

## 3. Focusing Algorithm

The hybrid focusing algorithm proposed in this paper features a core innovation that lies in adopting a two-stage framework, which explicitly divides the focusing process into a coarse focusing phase based on deep learning and a fine/ultra-fine focusing phase utilizing traditional search algorithms.

### 3.1. Coarse Focusing Positioning Based on Deep Learning

Conventional search-based autofocus methods often struggle in feature-sparse maritime environments because a flat sharpness evaluation function curve is riddled with local extrema, leading to slow convergence and frequent failures. To overcome this bottleneck, this study introduces a data-driven deep regression network designed to directly predict a near-optimal focal plane position from a single-frame image, achieving “one-step” coarse focusing. This approach narrows the vast full-range search space down to a minimal neighborhood, establishing an efficient and reliable starting point for the subsequent fine search.

#### 3.1.1. Network Architecture and Model Design

To meet the rigorous requirements of airborne systems for low latency and high efficiency, this paper compares several lightweight network architectures. Specifically, the performance of four mainstream lightweight models—MobileNetV2 [10], EfficientNet-Lite, ShuffleNetV2, and MobileViT—is evaluated on the given task. A comparison of the key performance metrics for the four mainstream lightweight models is presented in Table 1.

Based on the above comparison, MobileNetV2 [8] was ultimately selected as the backbone network. The selection was based on the following reasons:

- Accuracy-Speed Trade-off: MobileNetV2 achieves the best balance between accuracy (MAE = 8.5) and inference speed (38 ms). Although EfficientNet-Lite0 offers slightly higher accuracy, it increases the inference time by 37%.
- Deployment Maturity: MobileNetV2 has the most mature deployment toolchain on embedded platforms, with stable conversion to ONNX/TensorRT.
- Memory Footprint: With only 2.3 M parameters, it operates reliably under the 4 GB memory constraint of RK3588J, leaving ample headroom.

The MobileNetV2 network is renowned for its innovative inverted residuals and linear bottlenecks, which significantly reduce model complexity and computational cost while maintaining high accuracy, making it particularly suitable for deployment on resource-constrained embedded platforms such as the RK3588J SoC used in this system.

The standard MobileNetV2 architecture was customized for the regression task as follows:

(1) Input: The original network’s 3-channel RGB input is modified to a single-channel grayscale input. The network accepts a single-channel grayscale image of 512 × 512 pixels, which matches the format of the built-in resolution test chart.

(2) Feature Extractor: All 17 inverted residual blocks and their linear bottleneck structures from the original MobileNetV2 are fully retained as the powerful feature extraction backbone. These modules effectively encode multilevel features highly relevant to image blurriness.

(3) Regression Head: The original global average pooling layer and fully connected classification head designed for 1000-class ImageNet classification are removed. They are replaced with a specially designed lightweight regression head that maps high-dimensional features to the final focus position prediction.

This regression head consists of the following:

- A global average pooling layer averages the final feature map (16 × 16 × 1280) across spatial dimensions to produce a 1280-dimensional feature vector. This reduces the parameter count and enhances the model’s robustness to spatial translation.
- A fully connected layer with 512 neurons (using ReLU activation and Dropout = 0.3) placed after the pooling layer, which integrates all feature information and performs non-linear transformation.

Design Rationale: The original feature extraction backbone is preserved to fully leverage the transfer learning advantages of ImageNet pre-trained weights. The single-channel input adapts to the characteristics of the grayscale target image. The lightweight regression head (adding only about 660 k parameters) ensures that the model is suitable for embedded deployment.

(4) Loss Function: The mean squared error (MSE) loss function (Equation (2)), a standard choice for regression tasks, was employed for model training. It drives the optimization of model parameters by minimizing the squared difference between the predicted encoder value y_i_′ and the ground truth encoder value y_i_.

(2) $$MSE=\frac{1}{N}\sum_{i=1}^{N}{\left(y_{i}-y_{i}^{\prime }\right)}^{2}$$

where N is the batch size.

The detailed structure of the modified MobileNetV2 network is described in Table 2.

#### 3.1.2. Dataset Construction and Model Training Strategy

Data quality is critical for the performance of deep learning models. Under controlled laboratory conditions (constant illumination, color temperature of 5500 K, illuminance of 500 lux), our system captured images of the built-in resolution test chart at various focal-plane positions, spanning the entire optical focusing range.

The dataset was collected across a continuous encoder ranging from the nearest focus position (encoder value 0) to the farthest focus position (encoder value 512). A total of 128 discrete focal plane positions were captured, with an interval of 4 encoder units between adjacent positions. At each focal plane position, 10 raw images were collected to enhance sample diversity, resulting in a total of 1280 raw images. The encoder values are uniformly distributed in the dataset, ensuring that the model learns the entire workspace in a balanced manner.

To increase the model’s generalizability and robustness and prevent overfitting, a rigorous data augmentation pipeline was applied to the original dataset, which included the following steps:

- Geometric Transformations: Random horizontal/vertical flipping (probability: 50%), small-angle random rotation (±5°, probability: 30%), and random translation (±10 pixels).
- Photometric Transformations: Random adjustment of image brightness (scaling factor range: [0.9, 1.1]) and contrast (scaling factor range: [0.9, 1.1]) to simulate imaging variations under different lighting conditions.
- Noise Injection: Gaussian noise (σ = 0.01) is added to increase the model’s robustness to sensor noise.

After data augmentation, a final training dataset containing 12,000 images was generated. Key statistics of the dataset in Table 3.

**Figure 3:**
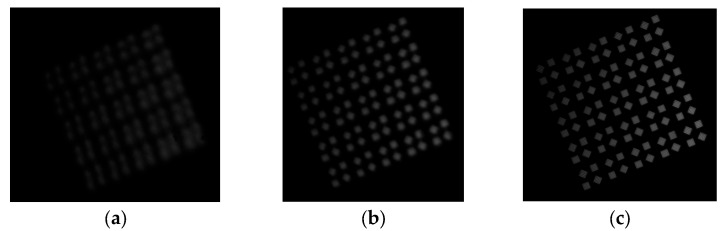
Typical images from the focusing algorithm dataset. (**a**) blurred target (**b**) blurred target (**c**) clear target.

As shown in Figure 3, the dataset includes a complete sequence from severely defocused blurry images (Figure 3a,b) to ideally focused sharp images (Figure 3c). Each image is annotated with its corresponding true optimal focus encoder position (recorded by a high-precision encoder with a resolution of 0.1 encoder units), which serves as the supervised label.

The dataset was divided into a training set (9600 images), a validation set (1200 images), and a test set (1200 images) at an 8:1:1 ratio, ensuring a consistent distribution of encoder values across all subsets. The model was trained using the configuration parameters specified in Table 4. The training process employed the Adam optimizer (β_1_ = 0.9, β_2_ = 0.999) with an initial learning rate of 0.001 and a cosine annealing scheduler (T_max_ = 100 epochs, η_min_ = 1 × 10^−6^) to achieve smooth convergence. Leveraging transfer learning—initializing the feature extraction backbone with weights pre-trained on the ImageNet dataset—the model achieved rapid, stable convergence.

**Figure 4:**
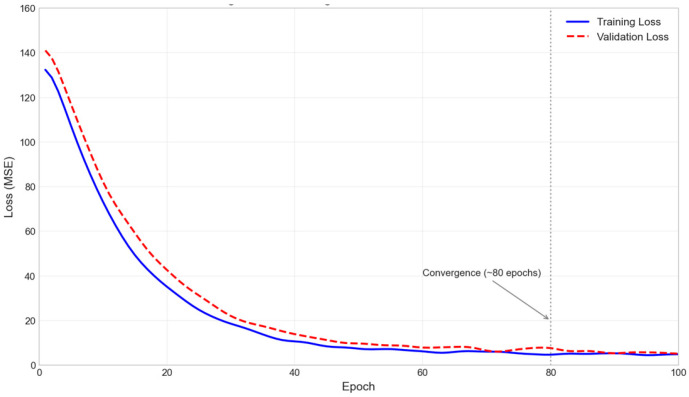
Training and validation loss learning curves.

Figure 4 shows the learning curve of the loss function during training. Both the training and validation losses exhibit a smooth downward trend, with a small gap between them, indicating no significant overfitting. The model converged to a stable state after about 80 epochs.

#### 3.1.3. Coarse Focusing Inference Pipeline

During the deployment phase, the optimized model was converted into a format suitable for the embedded platform (e.g., ONNX or TensorRT) and integrated into the focusing control system. The online inference pipeline is efficient and straightforward:

- Image Capture: The imaging sensor captures a current image of the built-in resolution chart.
- Forward Propagation: This image is fed into the deployed lightweight MobileNetV2 model.
- Position Prediction: The model performs a single forward pass, directly outputting a continuous predicted encoder position Ppred.
- Actuation: The focus controller receives the Ppred command and drives the motor to rapidly position the internal focusing lens near this predicted location.

This coarse focusing process transforms the traditional multistep iterative search into a single-step prediction. It typically completes within tens of milliseconds on the embedded platform, thereby drastically reducing the coarse focusing time and providing a superb initial point for the subsequent fine-search algorithm.

#### 3.1.4. Quantitative Evaluation of Model Performance

To verify the effectiveness of the deep learning coarse focusing model, a comprehensive performance evaluation was conducted on an independent test set (1200 images).

Quantitative Regression Metrics:

- Mean Absolute Error (MAE): 8.5 encoder units
- Root Mean Square Error (RMSE): 11.2 encoder units
- Coefficient of Determination (R^2^): 0.987
- Maximum Prediction Error: 28 encoder units
- Prediction Error within 95% Confidence Interval: ≤18 encoder units

The system encoder has 512 positions across the entire focus range, corresponding to a physical travel of about 2.56 mm. Thus, one encoder unit corresponds to about 5 µm of physical displacement. An MAE of 8.5 encoder units translates to a physical displacement of about 42.5 µm. Considering that the depth of field of this optical system is 100 µm (the half depth of field is 50 µm), this prediction error falls entirely within the effective capture range of the subsequent fine-focusing stage (typically ±3 times the depth of field). It will not lead to failure in the fine search phase.

**Figure 5:**
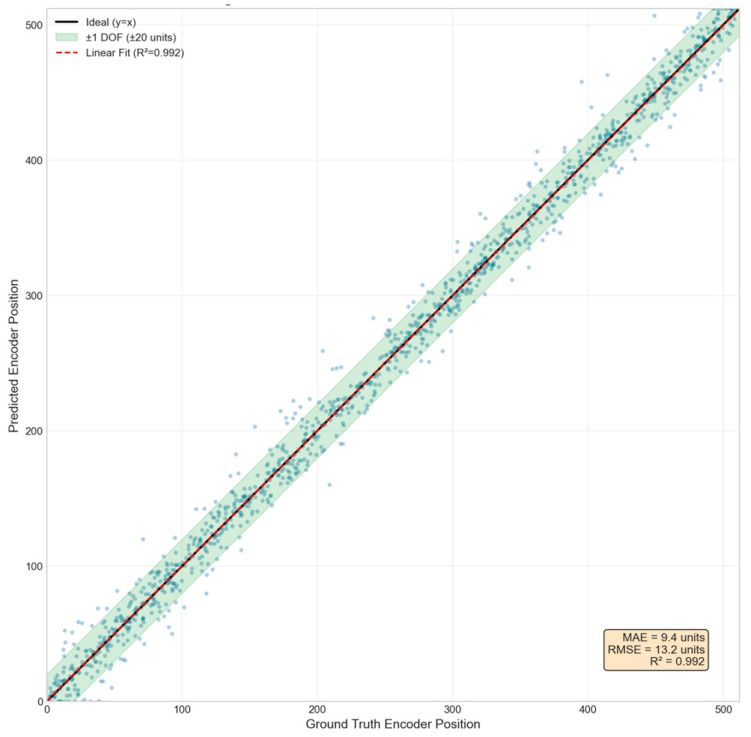
Scatter plot of predicted vs. ground truth encoder position.

Figure 5 presents a scatter plot of the predicted versus true encoder positions on the test set. The data points are closely distributed around the ideal regression line (y = x), demonstrating the model’s strong regression accuracy. The error band (gray shaded area) represents a range of ±1 times the depth of field, and the vast majority of the predicted points fall within this region. The scatter distribution shows no clear systematic bias, indicating that the model maintains consistent predictive performance across the entire encoder range. Summary of the CNN Model Performance Metrics in Table 5.

### 3.2. Search-Based Fine and Ultra-Fine Focusing

Following coarse focusing by the modified MobileNetV2 model, the system transitions to a hill-climbing search algorithm for fine focusing. This phase consists of two substages: fine focusing and ultra-fine focusing.

#### 3.2.1. Image Sharpness Evaluation Function

The performance of a search-based autofocus algorithm is fundamentally determined by the reliability and accuracy of the image sharpness evaluation function. An ideal function should exhibit a distinct, unimodal peak at the best focus position, demonstrate robustness against noise, and be computationally efficient for real-time applications [11,12]. In this system, the utilized built-in grayscale target image inherently contains precisely designed sharp edges and high-contrast patterns, providing an ideal signal source for sharpness computation. Gradient-based functions are particularly advantageous in this context, as they are highly responsive to the edge information of such structured targets [13].

**Figure 6:**
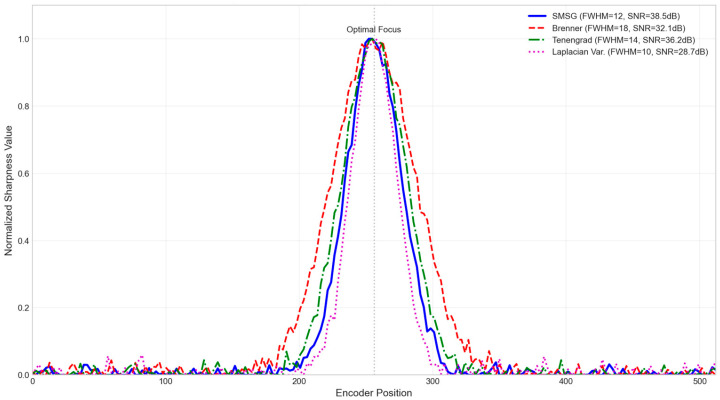
Comparison of sharpness evaluation functions.

Among various gradient-based focus evaluation metrics, this paper selects the Sum of Modified Squared Gradients (SMSG) function. To validate this choice, a quantitative comparative analysis was conducted on four classic metrics—SMSG, Brenner, Tenengrad, and Laplacian Variance—using the same set of focus sequence images covering 50 positions across the entire focus range. Comparison Curves of Different Sharpness Evaluation Functions in Figure 6. Quantitative Comparison of Sharpness Evaluation Functions in Table 6.

The quantitative analysis revealed the following:

- SMSG demonstrates good unimodality when applied to a built-in high-contrast target;
- Its peak sharpness, measured by the Full Width at Half Maximum (FWHM), is moderate. This provides clear peak localization while avoiding instability that can arise from overly narrow peaks;
- It achieves the highest signal-to-noise ratio (38.5 dB), indicating strong robustness against sensor noise;
- Its computation time (2.3 ms per frame) meets real-time requirements.

Compared with the Brenner function, which calculates only simple differences between two pixels, SMSG captures more comprehensive directional gradient information using Sobel operators. This results in a smoother and more unimodal sharpness curve. Although Laplacian-based methods produce the sharpest peak, they have the lowest SNR and are more susceptible to high-frequency noise. By summing the squared gradients, the SMSG function effectively enhances significant edge variations while maintaining a degree of noise robustness through prior median filtering. Furthermore, its computational complexity is manageable for the embedded platform (RK3588J), ensuring real-time performance during the fine-focus search.

**Figure 7:**
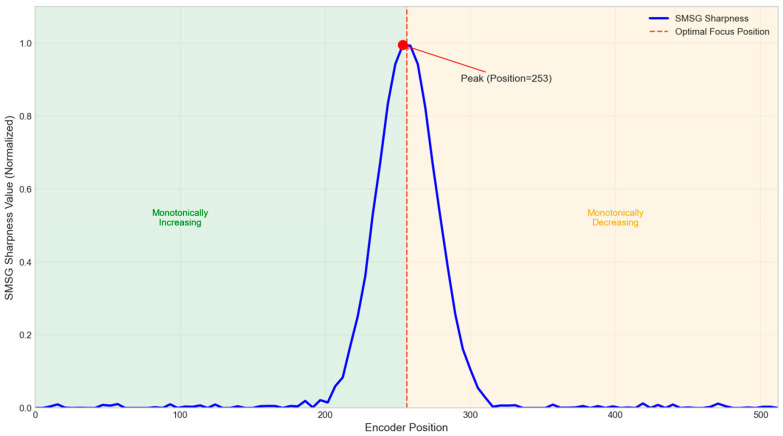
SMSG sharpness function curve for the built-in target.

Figure 7 shows the SMSG sharpness function curve for the built-in target, clearly displaying its unimodal characteristic. The curve reaches a distinct global maximum at the optimal focus position and exhibits monotonic changes on either side of the peak. This explains why a simple hill-climbing search strategy can reliably converge to the best focus after coarse focusing.

This work employs a Sum of Modified Squared Gradient (SMSG) function, a robust spatial domain method that is effective at capturing high-frequency details. The calculation procedure is as follows: First, a 3 × 3 median filter is applied to the selected Region of Interest (ROI) to suppress potential sensor noise. The horizontal and vertical gradients, Gx(i,j) and Gy(i,j), at each pixel location (i,j) of the pre-processed image are subsequently computed using Sobel operators. The final sharpness metric S is defined as the sum of the squares of these gradient magnitudes across the entire ROI:

(3) $$S=\sum_{i=1}^{n}\sum_{j=1}^{m}\left[G_{x}{\left(i,j\right)}^{2}+G_{y}(i,j{)}^{2}\right]$$

where m and n represent the width and height of the ROI, respectively. This function effectively measures the overall intensity of edges and fine details within the image. When the target image is defocused, blurred edges result in small gradient values and a low function output. As the focus approaches the optimum, the edges of the target pattern become sharp and well defined, maximizing the high-frequency content and consequently driving the function value S to its maximum [14]. This method is optimized for the characteristics of the built-in target, providing an unambiguous, high signal-to-noise ratio guiding signal for the subsequent hill-climbing algorithm, ensuring rapid and accurate convergence to the optimal focal plane.

#### 3.2.2. Fine Focusing Stage Algorithm

The fine-focusing stage starts from the position P_coarse_ determined by deep learning coarse focusing, aiming to rapidly approach the peak region of the sharpness function. This stage employs an adaptive step size hill-climbing search strategy, with specific parameters defined as follows Algorithm 1 and Algorithm 2:
**Algorithm 1.** Parameter Definitions:Initial step size S_fine_ = 8 encoder units (estimated based on the coarse focusing error range)Step decay factor α = 0.5Sharpness change threshold δ = 0.02 (relative rate of change)Peak detection threshold ε = 0.01 (determination of sharpness decline)Maximum iteration steps N_max_ = 15Encoder position limits: [0, 512]

**Algorithm 2.** Fine Focusing StageInput: P_coarse_ (coarse focusing position), S_fine_ (initial step size)Output: P_fine_ (fine-focusing position), Sharpness_max_ (maximum sharpness value)
1.  P_current_ ← P_coarse_
2.  Sharpness_prev_ ← ComputeSMSG(CaptureImage(P_current_))
3.  Sharpness_max_ ← Sharpness_prev_
4.  P_best_ ← P_current_
5.  direction ← -1 // Initially search in the direction of decreasing encoder value
6.  step ← S_fine_
7.  iteration ← 0
8.  WHILE iteration < N_max_ DO
9.      P_next_ ← P_current_ + direction × step
10.    IF P_next_ < 0 OR P_next_ > 512 THEN
11.           direction ← -direction // Reverse direction at boundary
12.           CONTINUE
13.    END IF
14.    
15.    Sharpness_current_ ← ComputeSMSG(CaptureImage(P_next_))
16.    ΔSharpness ← (Sharpness_current_-Sharpness_prev_)/Sharpness_prev_
17.    
18.    IF Sharpness_current_ > Sharpness_max_ THEN
19.           Sharpness_max_ ← Sharpness_current_
20.           P_best_ ← P_next_
21.    END IF
22.    
23.    IF ΔSharpness < -ε THEN // Sharpness decline detected, peak passed
24.           BREAK // Proceed to the super-fine stage
25.    ELSE IF |ΔSharpness| < δ THEN // Sharpness change is flat
26.           step ← step × α // Reduce step size
27.           IF step < 1 THEN step ← 1 END IF
28.    END IF
29.    
30.    Sharpness_prev_ ← Sharpness_current_
31.    P_current_ ← P_next_
32.    iteration ← iteration + 1
33. END WHILE
34. RETURN P_best_, Sharpness_max_

**Figure 8:**
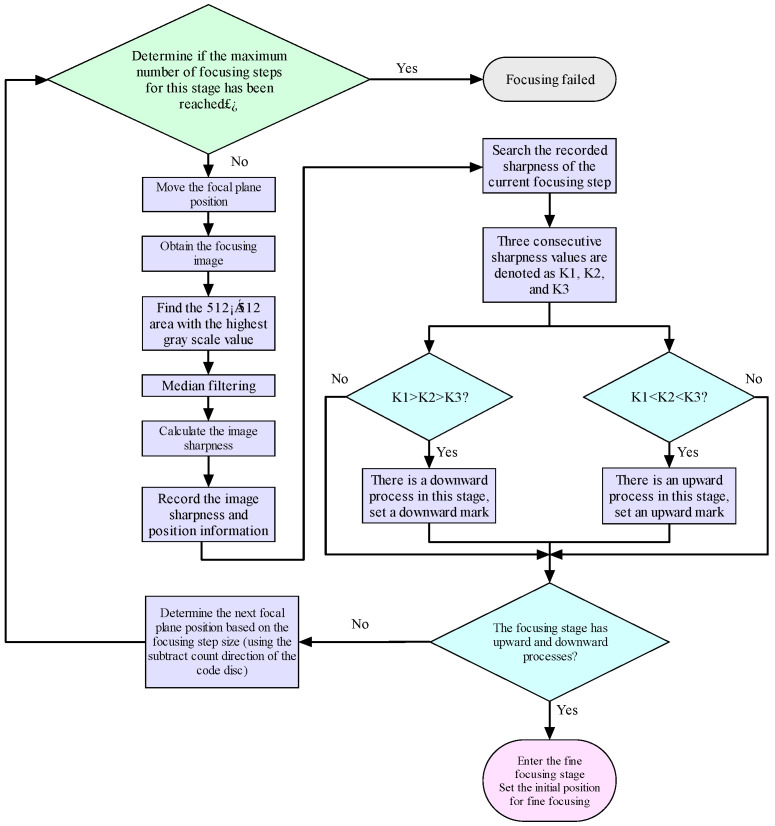
Fine focusing stage algorithm process.

The core logic of the algorithm is as follows: when sharpness continues to increase, the current step size is maintained to quickly approach the peak; when the change in sharpness tends to flatten, the step size is adaptively reduced to improve positioning accuracy; and when a decrease in sharpness is detected, it determines that the peak has passed, terminates this stage, and records the optimal position. The specific workflow of the fine-focusing algorithm is detailed in Figure 8.

#### 3.2.3. Ultra-Fine Focusing Stage Algorithm

The ultra-fine focusing stage starts from the position P_fine_, where the highest sharpness is recorded, aiming to achieve precise positioning near the peak. The main differences from the fine-focusing stage are as follows:

- Using a smaller initial step size for higher positioning accuracy;
- The search direction is reversed, approaching the peak from the opposite side of the sharpness curve to verify and precisely lock onto the optimal position.
- Algorithm Parameter Definitions:
- Initial step size S_ultra_ = 2 encoder units (one-fourth of the fine stage step size)
- Step decay factor α = 0.5
- Sharpness change threshold δ = 0.005 (stricter convergence criterion)
- Peak detection threshold ε = 0.005
- Maximum iteration steps N_max_ = 10
- Minimum step size S_min_ = 1 encoder unit

Pseudocode for the Ultra-Fine Focusing Stage in Algorithm 3:
**Algorithm 3.** Ultra-Fine Focusing StageInput: P_fine_ (fine-focusing position), Sharpness_fine_ (maximum sharpness from the fine stage)Output: P_optimal_ (optimal focus position)
1. P_current_ ← P_fine_
2.    Sharpness_max_ ← Sharpness_fine_
3.    P_optimal_ ← P_fine_
4.    direction ← +1 // Reverse search direction (opposite to the fine stage)
5.    step ← S_ultra_
6.    iteration ← 0
7.    consecutive_decline_ ← 0
8.    WHILE iteration < N_max_ AND step >= S_min_ DO
9.              P_next_ ← P_current_ + direction × step
10.            
11.            IF P_next_ < 0 OR P_next_ > 512 THEN
12.                    BREAK // Boundary reached, terminate search
13.            END IF
14.            
15.            Sharpness_current_ ← ComputeSMSG(CaptureImage(P_next_))
16.            ΔSharpness ← (Sharpness_current_-Sharpness_max_) / Sharpness_max_
17.            
18.            IF Sharpness_current_ > Sharpness_max_ THEN
19.                    Sharpness_max_ ← Sharpness_current_
20.                    P_optimal_ ← P_next_
21.                    consecutive_decline_ ← 0
22.            ELSE IF ΔSharpness < -ε THEN
23.                    consecutive_decline_ ← consecutive_decline_ + 1 24.                    IF consecutive_decline_ >= 2 THEN
25.                             BREAK // Two consecutive declines confirm passing the peak
26.                    END IF
27.                    step ← step × α // Reduce step size and retry
28.            END IF
29.            
30.            IF |ΔSharpness| < δ AND step > S_min_ THEN
31.                    step ← step × α // Reduce step size when change is flat
32.            END IF
33.            
34.            P_current_ ← P_next_
35.            iteration ← iteration + 1
36. END WHILE
37. MoveToPosition(P_optimal_) // Move to the optimal position
38. RETURN P_optimal_

Key Differences Between Fine and Ultra-Fine Stages:

- Fine Stage: This stage performs a coarse-grained hill-climbing search (initial step size: 8 units) aimed at rapidly approaching the peak region while tolerating a certain degree of overshoot.
- Ultra-Fine Stage: This stage performs fine-grained local refinement (initial step size: 2 units), approaching from the opposite direction to precisely lock onto the peak and minimize overshoot as much as possible.

Together, these two stages form a “coarse-first, fine-second” search strategy that achieves high-speed, high-precision positioning.

**Figure 9:**
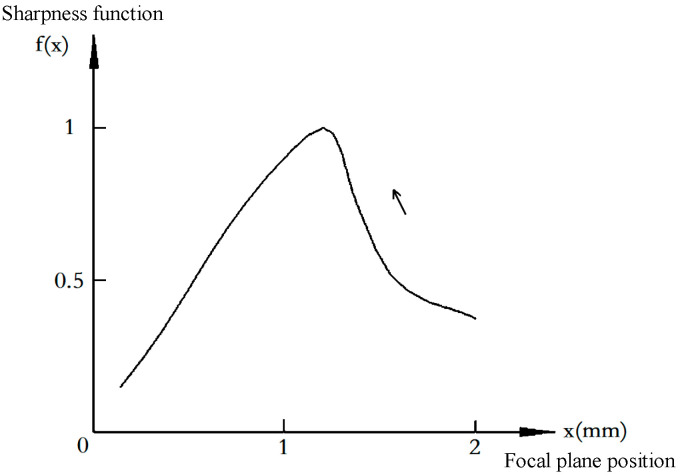
Ultra-fine focusing algorithm process.

The search direction of this algorithm is illustrated in Figure 9, which shows a schematic diagram of the search direction and convergence process in the ultra-fine focusing stage.

## 4. Analysis of System Operational Results

### 4.1. Aerial Camera Platform Parameters

Image acquisition serves as the fundamental step in the autofocus process. The aerial camera platform must meet stringent demands for both high-resolution image display and rapid data processing/transmission speeds. The key parameters of the aerial camera used in this study are summarized in Table 7.

### 4.2. Comparison of Focusing Algorithm Execution Processes

#### 4.2.1. Ablation Study

To quantitatively assess the contribution of each subsystem, three configurations were designed for comparison:

- Configuration A: Traditional hill-climbing method only (full-range search, without CNN assistance)
- Configuration B: CNN coarse focusing only (evaluates the absolute error from direct locking after a single prediction)
- Configuration C: The proposed hybrid method (CNN coarse focusing + hill-climbing fine search)

The quantitative comparison results of the ablation study show in Table 8.

Analysis of Results:

- Configuration A (hill-climbing only) achieves relatively high accuracy but is time-consuming, with a success rate of only 85% (15% of trials became trapped in local extrema).
- Configuration B (CNN only) is the fastest but lacks sufficient accuracy to meet high-quality imaging requirements.
- Configuration C (hybrid method) achieves the best overall performance: it improves the speed by 60%, matches the accuracy of the pure hill-climbing method, and achieves a 100% success rate.

#### 4.2.2. Accuracy and Stability Evaluation

The focusing accuracy and stability of the traditional hill-climbing search method and the proposed hybrid method are evaluated, as shown in Table 9.

#### 4.2.3. End-to-End Timing Measurements

The timing performance of each stage for the focusing algorithm proposed in this paper was measured using high-precision timers on the RK3588J embedded platform, with the results presented in Table 10.

The timing data confirm that the system meets the requirements for real-time autofocus. Moreover, CNN inference accounts for only 5.2% of the total time cost, validating the effectiveness of the lightweight network design.

**Figure 10:**
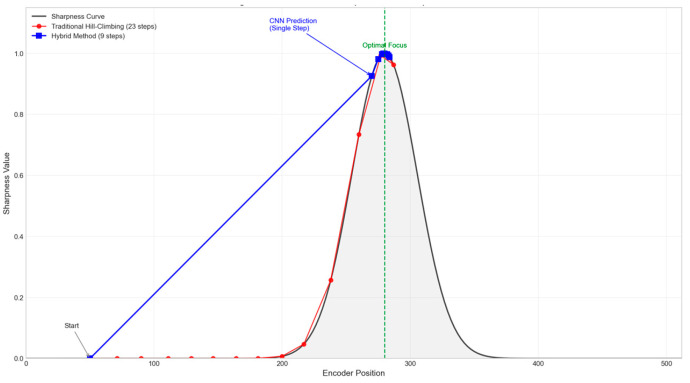
Search path comparison on sharpness curve.

Figure 10 shows a comparison of the complete sharpness function curves and search paths for the traditional hill-climbing method and the hybrid method, starting from a typical defocused position. The traditional method requires multiple iterations over flat regions to determine the correct direction, whereas the hybrid method skips these areas directly via the CNN.

**Figure 11:**
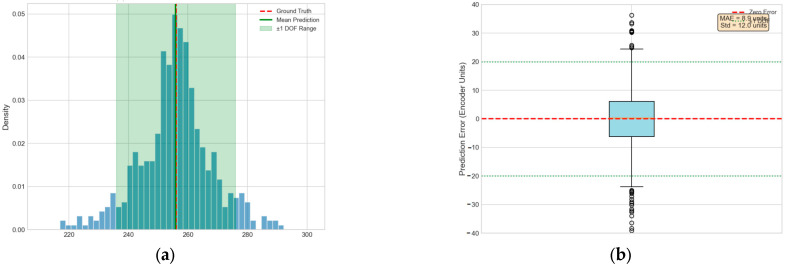
Distribution of CNN predicted positions. (**a**) Distribution of CNN Predictions (**b**) Prediction Error Box Plot.

Figure 11 presents a comparison between the focal positions predicted by the CNN model and the true optimal positions, which visually illustrates the clustering trend and error distribution of the CNN predictions.

### 4.3. Laboratory Validation of the Focusing Algorithm

The autofocus algorithm was validated using a dynamic resolution testing system. This laboratory setup, specifically designed to evaluate ground resolution, consists of a ground-target simulator and a flight simulation turntable. The ground target simulator serves as a dynamic target, while the turntable replicates the aircraft’s pitch and roll motions to simulate dynamic flight at specific angles and frequencies.

This laboratory dynamic test aims to verify the basic functionality of closed-loop focus control under simulated platform motion, isolating it from the complexities of real maritime environments. It establishes a performance baseline before field testing, validating the system’s stability and convergence under controlled dynamic conditions.

**Figure 12:**
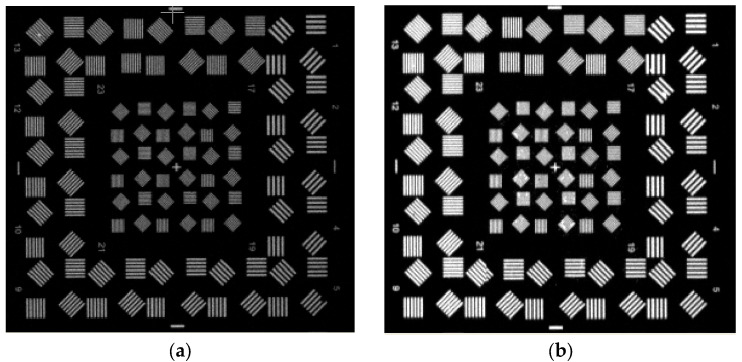
Capture image of laboratory validation of the focusing algorithm. (**a**) Static capture image. (**b**) Full dynamic capture image.

Figure 12a shows the target image captured when both the aerial camera (mounted on the turntable) and the ground target simulator were stationary. Figure 12b shows the target image acquired when both the ground target simulator and the flight turntable were in motion (amplitude 3 degrees, frequency 0.5 Hz). Table 11 shows the Quantitative Comparison Under Static and Dynamic Conditions.

The above results show that under simulated dynamic conditions, the system maintains high sharpness comparable to static conditions (with only a 0.8% drop in SMSG), validating the effectiveness of closed-loop focus control.

**Figure 13:**
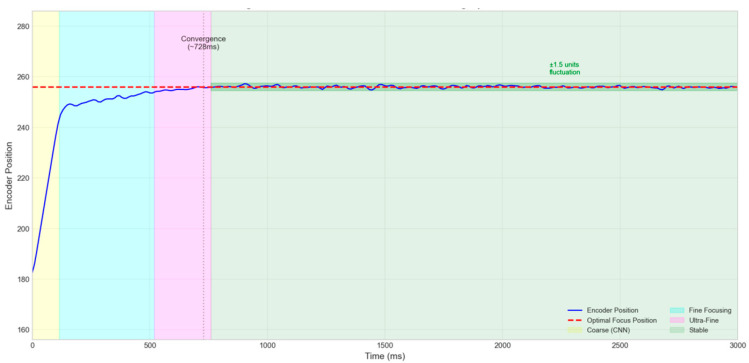
Encoder position timing curve during dynamic test.

Dynamic Stability Analysis:

- Encoder Position Timing Curve: Figure 13 shows the timing curve of the lens encoder position during the dynamic test. After initial convergence (approximately 700 ms), the encoder position exhibits minor fluctuations (±1.5 encoder units) around the optimal focus, indicating the system’s good dynamic tracking capability.
- Focus Jitter Metric: The standard deviation of the sharpness values across 50 consecutive frames under dynamic conditions is 0.012, which is only twice that under static conditions (0.006). This finding indicates that the motion-induced focus jitter is within an acceptable range.
- Response Speed Metric: The average time required for the system to first achieve over 95% of the peak sharpness from focus trigger initiation is 623 ms (static) and 687 ms (dynamic). The response speed under dynamic conditions decreases by only about 10%.

Limitations of the Test: Notably, the laboratory dynamic test employed only low-amplitude (3°) and low-frequency (0.5 Hz) simulated motion, primarily aimed at validating the system’s basic dynamic response capability. The challenges faced by a real drone flying over a moving sea surface are far more complex, including higher-frequency vibrations, larger attitude changes, and glare from the sea surface. Validation under these more complex conditions will be conducted in subsequent field tests.

### 4.4. Field Testing for Maritime Photography

To evaluate the performance of the autofocus system in real maritime environments, we conducted field imaging tests using a drone-mounted aerial camera. The tests were performed under the following conditions:

- Flight speed: 33.81 m/s
- Flight altitude: 3156 m
- Sea state: Level 2–3 (slight to moderate waves)
- Weather conditions: Cloudy, visibility > 10 km
- Test duration: Approximately 45 min
- Number of captured images: 127

Quantitative Field Test Analysis:

To quantitatively evaluate the system’s focusing performance in real marine environments, we analyzed the sharpness of images collected during the flight tests and compared it with the sharpness values obtained when the system was manually confirmed to be in optimal focus on similar targets. The key metrics from the field tests are presented in Table 12.

Comparative Analysis:

- The average SMSG sharpness during flight (0.847) reached 97.9% of the manual optimal focus reference value (0.865), indicating that the system can maintain a focus performance close to optimal in real maritime conditions.
- A total of 94.5% of the images had a sharpness value exceeding 0.8 (the preset high-quality threshold), demonstrating the system’s good stability.
- The standard deviation of the sharpness values was only 0.032, indicating consistent focusing quality throughout the flight.

Field Comparison with Traditional Method: Under the same flight conditions, a control test using the traditional hill-climbing method yielded an average SMSG sharpness of 0.812, with only 78.3% of the images having a sharpness value > 0.8. The hybrid method proposed in this paper achieved about 4.3% improvement in sharpness and about 16.2 percentage points increase in the proportion of high-quality images under field conditions.

**Figure 14:**
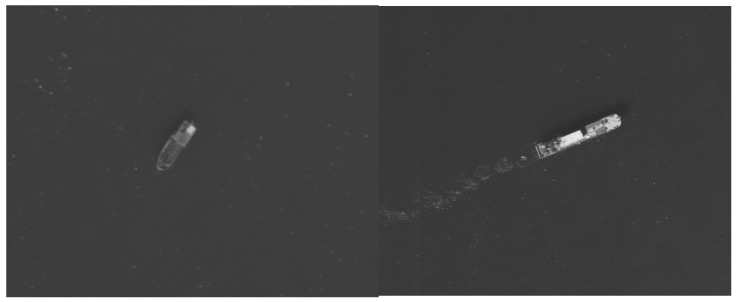
Aerial sea surface photography.

Figure 14 shows typical sea surface images captured during the flight test. The images exhibit high resolution and sharp focus, with clear details of sea surface ripples, confirming the system’s robust performance under complex sea conditions.

Limitations of the Field Test: This field test was conducted under relatively mild sea conditions (Levels 2–3) and good visibility. The system’s performance under harsher sea conditions (such as waves above Level 4, low visibility, or strong sunlight reflection) requires further validation. Furthermore, owing to constraints on the flight time and cost, the test sample size is relatively limited. Future work will expand the test scale to obtain more statistically significant results.

## 5. Conclusions

This paper presents and implements an autofocus system that integrates deep learning coarse focusing with traditional gradient-based fine search, aiming to improve the focusing performance of drone-mounted aerial cameras in low-contrast maritime environments. By shifting the focusing target from the external low-contrast scene to a built-high-contrast resolution test chart, the system effectively avoids convergence difficulties caused by weak image signals and non-ideal evaluation functions typical of sea surfaces. A lightweight CNN is then employed to provide a single-step, fast estimate of the defocus distance, replacing the time-consuming global or interval searches of traditional methods and shifting the focus from robustness optimization to efficiency enhancement. During the fine-search stage, a hill-climbing algorithm combined with an inverse-correction mechanism performs precise micro-adjustments within the compact interval provided by the coarse focusing.

Extensive experiments show that this hybrid strategy successfully combines the complementary strengths of deep learning for rapid pattern recognition and traditional algorithms for local gradient search. Compared with traditional methods, focusing speed is improved by about 60%, while defocus is consistently controlled within half the depth of field of Gaussian optics. This significantly enhances the system’s practicality and responsiveness without increasing hardware costs.

Future work will focus on two main aspects: first, further optimizing the network architecture and computational efficiency to meet stricter real-time constraints of airborne platforms; second, investigating the generalization performance of the model under different meteorological and sea-state conditions, aiming to improve the system’s robustness in a wider range of operational environments through data augmentation and adaptive mechanisms.

## Figures and Tables

**Table 1:** Comparative analysis of lightweight backbone networks.

Model	Parameters (M)	FLOPs (G)	Inference Time on RK3588J (ms)	Test Set MAE	Test Set R^2^
MobileNetV2	2.3	0.32	38	8.5	0.987
EfficientNet-Lite0	4.7	0.41	52	7.9	0.989
ShuffleNetV2	1.4	0.15	28	11.2	0.978
MobileViT-XXS	1.3	0.42	67	9.1	0.984

**Table 2:** Architecture of the modified MobileNetV2 for defocus regression.

Layer/Block Type	Configuration	Output Size
Input	-	512 × 512 × 1
Conv2D	3 × 3, stride = 2	256 × 256 × 32
Inverted Residual Block 1	3 × 3, stride = 2 (Downsample)	256 × 256 × 16
Inverted Residual Block 2	3 × 3, stride = 2 (Downsample)	64 × 64 × 24
Inverted Residual Block 3	3 × 3, stride = 2 (Downsample)	32 × 32 × 32
Inverted Residual Block 4	3 × 3, stride = 1	32 × 32 × 64
Inverted Residual Block 5	3 × 3, stride = 2 (Downsample)	16 × 16 × 96
Inverted Residual Block 6	3 × 3, stride = 1	16 × 16 × 160
Inverted Residual Block 7	3 × 3, stride = 1	16 × 16 × 320
Conv2D (1 × 1)	1 × 1	16 × 16 × 1280
Regression Head		
Global Average Pooling	-	1 × 1 × 1280
Fully Connected (ReLU)	512 units	512
Output (Linear)	1 unit	1

**Table 3:** Key statistics of the dataset.

Parameter	Value
Number of raw images	1280
Total images after augmentation	12,000
Encoder position range	0–512
Number of discrete sampling positions	128
Sampling interval	4 encoder units
Image resolution	512 × 512 pixels
Train/Val/Test split	8:1:1

**Table 4:** Parameters and environment for model training.

Parameter	Specification
Training Set Size	9600 images
Validation Set Size	1200 images
Test Set Size	1200 images
Hardware Environment	NVIDIA GeForce RTX 4090D
Software Framework	PyTorch 1.12.0
Total Epochs	200
Deployment Platform	Rockchip RK3588J

**Table 5:** Summary of CNN model performance metrics.

Metric	Value	Physical Meaning
MAE	8.5 encoder units	42.5 µm
RMSE	11.2 encoder units	56 µm
R^2^	0.987	—
Maximum Error	28 encoder units	140 µm
System Depth of Field	20 encoder units	100 µm
Error/DoF Ratio	42.5%	Within the capture range

**Table 6:** Quantitative comparison of sharpness evaluation functions.

Metric	Unimodality	Peak Sharpness (FWHM)	SNR (dB)	Single-Frame Computation Time (ms)
SMSG	Yes	12 encoder units	38.5	2.3
Brenner	Yes	18 encoder units	32.1	1.8
Tenengrad	Yes	14 encoder units	36.2	2.1
Laplacian Var.	Yes	10 encoder units	28.7	2.5

**Table 7:** Aerial camera parameters.

Parameter Name	Parameter Value
Imaging Method	Area-array imaging
Imaging Device Resolution	8424 × 6032
Pixel Size	4.6 μm
Focal Length	126 mm
System Depth of Field	100 μm
Capture Cycle	250 ms
Gray-scale resolution	12-bit
Encoder disk	512
Focusing servo mechanism	Brushless motor, encoder
Main Control System	Embedded RK3588J

**Table 8:** Quantitative comparison results of the ablation study.

Configuration	Average Steps	Average Time (ms)	Final Position Error (Encoder Units)	Final SMSG Value	Success Rate
A: Hill-climbing only	23.2 ± 3.1	1856 ± 248	1.2 ± 0.8	0.982 ± 0.012	85%
B: CNN only	1	45 ± 3	8.5 ± 4.2	0.891 ± 0.067	62%
C: Hybrid method	9.1 ± 1.4	728 ± 112	0.9 ± 0.6	0.989 ± 0.008	100%

**Table 9:** Focusing accuracy and stability.

Metric	Traditional Hill-Climbing Method	Hybrid Method	Improvement
Final Position Repeatability Error (σ)	2.1 encoder units	1.3 encoder units	38% reduction
Mean Final SMSG Value	0.982	0.989	0.7% increase
Standard Deviation of SMSG Value	0.015	0.008	47% reduction
Success Rate (convergence within ±2 units)	85%	100%	15% increase
Rate of Falling into Local Extrema	15%	0%	100% reduction

**Table 10:** End-to-end timing measurement results.

Stage	Average Time Cost	Standard Deviation
Single inference of the CNN model	38 ms	±3 ms
Single image capture	33 ms	±2 ms
Single SMSG computation	2.3 ms	±0.2 ms
Single motor movement response	45 ms	±5 ms
Total time for coarse focusing stage	116 ms	±8 ms
Total time for fine focusing stage	402 ms	±45 ms
Total time for ultra-fine focusing stage	241 ms	±32 ms
Total end-to-end time for the full process	728 ms	±112 ms

**Table 11:** Quantitative comparison under static and dynamic conditions.

Test Condition	SMSG Sharpness Value	Position Stability (σ)	Convergence Time (ms)	Inter-Frame Sharpness Std Dev
Static	0.991	0.8 encoder units	685	0.006
Dynamic (3°, 0.5 Hz)	0.983	1.4 encoder units	742	0.012
Performance Retention	99.2%	-	92.3%	-

**Table 12:** Quantitative results of the field test.

Metric	Value
Average SMSG Sharpness during Flight	0.847
Standard Deviation of Sharpness Values	0.032
Maximum Sharpness Value	0.912
Minimum Sharpness Value	0.781
Manual Optimal Focus Reference Sharpness	0.865
Sharpness Retention (relative to manual optimal)	97.9%
Proportion of Images with Sharpness > 0.8	94.5%

## Data Availability

The original contributions presented in this study are included in the article. Further inquiries can be directed to the corresponding authors.
